# Beyond NK Cells: The Expanding Universe of Innate Lymphoid Cells

**DOI:** 10.3389/fimmu.2014.00282

**Published:** 2014-06-16

**Authors:** Marina Cella, Hannah Miller, Christina Song

**Affiliations:** ^1^Department of Pathology and Immunology, Washington University School of Medicine, St. Louis, MO, USA

**Keywords:** innate lymphoid cells, IL-22, NKp44, cytokines, mucosal tissues

## Abstract

For a long time, natural killer (NK) cells were thought to be the only innate immune lymphoid population capable of responding to invading pathogens under the influence of changing environmental cues. In the last few years, an increasing amount of evidence has shown that a number of different innate lymphoid cell (ILC) populations found at mucosal sites rapidly respond to locally produced cytokines in order to establish or maintain homeostasis. These ILC populations closely mirror the phenotype of adaptive T helper subsets in their repertoire of secreted soluble factors. Early in the immune response, ILCs are responsible for setting the stage to mount an adaptive T cell response that is appropriate for the incoming insult. Here, we review the diversity of ILC subsets and discuss similarities and differences between ILCs and NK cells in function and key transcriptional factors required for their development.

## Introduction

The adaptive immune system has evolved antigen receptor diversity to cope with a large variety of pathogens and non-self antigens. It is well-established that, depending on the nature of the invader and the signals coming from the surrounding environment, the adaptive immune system mounts a specific effector T helper (Th) response that serves to control the trespasser and re-establish homeostasis. Functionally, Th1 eliminate intracellular pathogens, Th2 are critical in promoting the eradication of helminthes, and Th17 control fungal and bacterial infections (1).

In the last few years, it has become evident that the innate immune system displays a similar strategy in order to ensure a first line of defense against intruders via innate lymphoid cells (ILCs). Broadly, ILCs are defined by their lymphoid lineage and their lack of RAG-mediated recombined antigen receptors. Mirroring the Th subsets, ILC populations diverge from one another in their reliance on unique transcription factors and production of signature cytokines. In general, ILCs provide primary border patrol at mucosal surfaces sensing changes in the microenvironment and rapidly responding to insults by producing specific cytokines that limit the damage caused by the attacking pathogen or favor its clearance and elimination.

Until ILCs came into the spotlight, conventional NK cells (cNKs) (i.e., blood circulating CD56^+^ cells in human and splenic, lymph node, and bone marrow NKp46^+^NK1.1^+^ cells in mouse) were the only innate immune cells known to respond to cytokines produced by antigen presenting cells (APCs), such as dendritic cells (DCs) and macrophages (2–4).

Natural killer (NK) cells can rapidly release IFN-γ in response to IL-12 and IL-18 and/or type 1 IFN, which are produced when viruses and bacteria infect or come into contact with APCs. Because of their capacity to immediately respond to cytokines, cNK cells can be viewed as the prototype of all ILC subsets. However, they have additional properties, such as cytotoxic ability, that set them apart from other ILCs and allow them to eliminate virally infected or tumor-transformed cells.

Here, we will review the lineage relationship between ILC subsets and cNKs based on key transcription factors that regulate their respective development as well as the current understanding their functional roles.

## ILC Heterogeneity and Functional Specialization

Innate lymphoid cells, like Th cells, are heterogeneous and currently grouped in three major subsets: ILC1, ILC2, and ILC3 (5).

### Group 1 ILCs

ILC1s produce the Th1 signature cytokine IFN-γ. They include cNK [reviewed in detail in Ref. (6, 7)] and several additional, recently described, subsets of IFN-γ-producing cells (8–10) (Table 1).

**Table 1:** **Emerging subsets of ILC1 and tissue-resident NKs**.

	CD127	IL-15 dependence	IL-7 dependence	TBX21 dependence	Eomes dependence	E4BP4 dependence	Human counterpart
siLP ILC1	+++	Yes	No	Yes	No	Partial	Most likely CD127^+^CD56^−^CD94^−^mucosal cells
Intraepithelial ILC1	−	Partial	No	Yes	TBD	Yes	CD56^+^NKp44^+^
							CD103^+^CD160^+^ cells in human tonsil and intestine
“Converted” or “plastic” ILC3	+++	No, but IL-15 can promote conversion	Yes	Yes	No	TBD	Mostly generated *in vitro*
cNKs (splenic, bone marrow, lymph nodes)	−	Yes	No	Yes	Yes	Yes	Most likely CD56im^dim^CD16^+^ blood NKs
Thymic NK cells	+++	Yes	Yes	Yes	Yes	Yes	Most likely CD56right^bright^CD16^−^blood NKs
Liver-resident CD49a^+^DX5^−^NKs/ILC1	−	Yes	TBD	Yes	No	No	TBD
Skin-resident CD49a^+^DX5^−^NKs	−	Yes	TBD	Yes	TBD	No	TBD
Uterus-resident CD49a^+^DX5^−^NKs	−	Yes	TBD	No	TBD	No	TBD
Salivary gland NKs	−	Yes	TBD	TBD	TBD	No	TBD

*TBD, to be determined*.

#### A brief overview of cNK cells

Conventional NK cells have been known and actively studied for almost four decades (11, 12). cNKs were first described as a cell subset capable of rapidly eliminating tumor-transformed or allogeneic cells without the need for prior sensitization and in the absence of RAG-recombined antigen receptor recognition (13, 14). Traditionally, cNK cells are thought to be critical in conferring protection from viral infections, such as CMV (15), and in the immunosurveillance of tumor-transformed cells (16, 17).

In humans, cNKs include the CD56^bright^CD16^−^ and the CD56^dim^CD16^+^ subsets present in peripheral blood. CD56^bright^ NK cells are specialized in IFN-γ secretion in response to DCs/macrophages-derived cytokines, such as IL-12 and IL-18 (18) or T cell-derived cytokines, such as IL-2 (19), a functional feature that place them close to other ILC1 subsets. CD56^dim^CD16^+^ NKs are specialized in cytotoxicity, since they can readily release lytic granules containing perforin and granzyme upon contact with sensitive targets. However, it has been shown that also CD56^dim^ NK cells can produce IFN-γ, although with a more rapid kinetic and in a less sustained fashion than CD56^bright^ NKs (20). In addition, it has been suggested that CD56^bright^ can differentiate into CD56^dim^ NKs upon activation (21, 22). CD56^bright^ cells produce additional monokines such as GM-CSF, TNF-α, IL-13, and IL-10, suggesting that they may exert an immunoregulatory function in specific circumstances (23). In addition, CD56^bright^ NKs express CCR7, CXCR3, and CD62L and they are thought to traffic to secondary lymphoid organs via high endothelial venules (HEVs) (24).

In mouse, cNK include mature (CD11b^high^CD27^low^) and immature (CD27^high^CD11b^low^) circulating splenic and bone marrow NKs (25), CD127^+^ (the IL-7 receptor α chain) IL-7-dependent thymic-derived NKs (26), and different subsets of tissue-resident NKs (12, 27) whose nature, function, and relationship to other emerging subsets of ILC1 we are just beginning to understand. Tissue NKs include an abundant population of salivary gland NKs, which are poorly cytotoxic and poor producers of cytokines (28, 29), liver-resident CD49a^+^ (VLA1) DX5^−^ NK cells, and skin- and uterus-resident NK cells (30). Thymic NKs for their ability to produce IFN-γ, TNF-α, and GM-CSF in response to IL-12 are thought to represent the murine counterpart of human CD56^bright^ NK cells. One unifying feature of all NK cell subsets, including thymic NKs (31), is their dependence on IL-15 and IL-15Rα for development, survival, and maintenance.

The major breakthrough in understanding the biology and function of cNK cells was the discovery of germ line-encoded cell surface receptors of the C-type lectin or of the immunoglobulin superfamily that deliver either inhibitory or activating signal to NK cells (3, 32–35). These receptors, some of which are clonally distributed, finely tune NK cytolytic ability and the capacity to release cytokines through recognition of MHC class I molecules or other counter-receptors specifically expressed by target cells upon infection, tumor transformation, or stress-related signals (36). The appreciation of the complex array of signaling receptors expressed by cNKs has challenged the idea that NK cells simply exert “natural” cytotoxicity, and has suggested that in addition to “missing self” (37), which leads to release of inhibition mediated by inhibitory receptors that recognize self MHC class I, NK cells have to integrate a complex network of clues, which depend on contact with the target cells and/or the surrounding microenvironment. Accordingly, recent work has suggested that engagement of cytokine receptors can influence NK cell recognition mediated by activating receptors, such as NKG2D (38). Importantly, expression of inhibitory and activating receptors on NK cells controls their education and “licensing” (39), as well as their expansion during responses to pathogens. This expansion generates a long lived-memory-type NK cell progeny that is more effective in clearing infections upon a second challenge (6, 40).

Thus cNK cells possess some features adaptive immune cells that maximize their capacity to eliminate intruders while avoiding self-reactivity (7).

#### Emerging ILC1 subsets

One first subset of ILC1 is present in human mucosal tissues and expresses CD127 and the C-type lectin CD161, but does not express other markers of the NK lineage such as CD94, CD56, NKp44 (NCR2), or NKp46 (NCR1); although it rapidly responds to IL-12 and IL-18 by producing IFN-γ (8). These cells also do not express c-kit (the receptor for stem cell factor, SCF), which marks many other ILC subsets.

The second subset was identified in human tonsillar tissue and is characterized by the expression of several NK-related markers such as CD56, NKp46, and NKp44 (9). However, this subset also expresses markers of tissue-resident memory CD8 T cells such as CD103, CD49a, and CD101. These ILC1s are also present in mouse small intestine, have an intraepithelial location, and are distinguished by the expression of CD160, a receptor of the immunoglobulin superfamily that binds to the TNF receptor superfamily member HVEM. HVEM–CD160 interactions promote epithelial integrity by inducing the alternative NF-kB signaling pathway and Stat3 phosphorylation in epithelial cells (41). Intraepithelial resident ILC1s are distinguished by cNKs because they do not respond to IL-12 and IL-18. Alternatively, they secrete large amounts of IFN-γ upon stimulation with IL-15. Notably, intraepithelial ILC1s are only partially dependent on IL-15/IL-15Rα signaling for their development, a feature that set them apart from cNKs. Interestingly, these ILC1s seem to promote tissue damage in a mouse model of colitis induced by CD40 ligation in immune-deficient mice. Although the function of the newly described human ILC1 subsets has not been investigated in detail *in vivo*, they may play important pathogenic roles in human inflammatory bowel diseases (IBDs) as both CD56^−^CD127^+^ and NKp44^+^CD103^+^ ILC1s are increased in patients with Crohn’s disease, as compared to control individuals (8, 9). For instance, earlier studies have suggested that IL-15 and retinoic acid are highly pathogenic factors in celiac disease, since they induce production of IL-12 by intestinal DCs (42). In light of the fact that one ILC1 subset is responsive to IL-12, while the other promptly reacts to IL-15, it would be interesting to explore the respective role of these ILC1 subsets in human celiac disease or mouse models of celiac disease.

A putative murine equivalent of the CD56^−^CD127^+^ human ILC1s has been recently described in small intestine lamina propria (siLP) (10). These cells are CD127^+^, but also express markers of the NK lineage, such as NKp46 and NK1.1. Interestingly, siLP ILC1s, as cNKs, depend on IL-15, but not IL-7, for their development. *In vivo* these cells are major producers of IFN-γ and TNF-α in response to oral infection with *Toxoplasma gondii (T. gondii)* and promote clearance of this pathogen by recruiting inflammatory monocytes through the CCR1/CCL3 axis (43). Therefore, in these settings, siLP ILC1s play a major protective role. Most likely, clearance of *T. gondii* requires a strong Th1 environment, while in Crohn’s disease a robust Th1 signature sustains autoimmune damage and tissue destruction, explaining why ILC1s may play protective or pathogenic roles in different scenarios and in different diseases.

Another subset of ILC1s is represented by the so-called “converted” or “plastic” ILC3s. These cells can be generated *in vitro* from ILC3s in response to cytokines such as IL-15, IL-2, IL-12, and IL-23 that induce IFN-γ production (8, 44). They can also be induced *in vivo* by transfer of RORγt^+^ ILC3s and visualized by fate-mapping experiments in siLP (10, 45, 46).

### Group 2 ILCs

ILC2s produce the Th2 signature cytokines IL-5 and IL-13 (47–50). They also produce amphiregulin and IL-9, and notably, IL-9/IL-9 receptor signaling is required for their survival (51). ILC2s are found in various tissues including adipose tissue-associated lymphoid structures, gut, lung (52) and, as recently described, the skin (53–55). They promote expulsion of parasites (56, 57) and maintain lung homeostasis (58) or drive airway hyper-reactivity during viral infections, such as influenza (59). They also contribute to the pathogenesis of atopic dermatitis (53–55). In visceral adipose tissue, ILC2s maintain metabolic homeostasis by recruiting eosinophils, which sustain macrophage alternative activation (60, 61). ILC2s rapidly respond to the alarmin IL-33, to the IL-17 family member IL-25 and to TSLP in skin (62). They are characterized by the expression of CD127, c-kit, Sca1, and ST2 (the receptor for IL-33). Human ILC2s express CD161 and high levels of the prostaglandin D2 receptor CRTH2 (63). Notably, ILC2s are highly enriched in nasal polyps of patients with chronic rhinosinusitis (63), suggesting that they might play a fundamental role in human Th2-mediated diseases such as asthma and atopic dermatitis. A subset of cells named multipotent progenitor type 2 (MPP^type2^), originally classified within ILC2s because of their ability to expand in response to IL-25, have been recently shown to contain progenitors giving rise to myeloid cells, such as macrophages and eosinophils (50). Because of the distinct transcriptional profile and functional potential that distinguishes MPP^type2^ from other ILC2s, and ILCs in general, these cells are no longer classified as Group 2 ILCs.

### Group 3 ILCs

ILC3s produce the Th17 signature cytokines IL-17 and/or IL-22 (64–69). ILC3s include Lineage^−^ (Lin)RORγt^+^ CD4^+^ Lti-like cells originally described in the 1990s (70), Lin^−^ RORγt^+^ CD4^−^ Lti-like cells, NCR^+^ ILC3s originally named NK-22 (65), and colonic Sca1^+^ Thy1^high^ ILCs (71). ILC3s rapidly respond to IL-23, a member of the IL-12 family. They also express the IL-1 receptor and respond to IL-1β (44, 72). ILC3s are mainly found in mucosal tissues, such as small and large intestine, Peyer’s patches (PP), and gut-associated lymphoid tissue (GALT). Small numbers of ILC3s are present in spleen (73) and lung (74). By producing IL-22, ILC3s protect intestinal epithelium *in vivo* from attaching and effacing bacteria, such as *Citrobacter rodentium*, a mouse model of human enteropathogenic *E. coli* (75). IL-22 acts selectively on stromal and epithelial cells by inducing STAT3 phosphorylation, leading to multiple downstream events including the rapid production of the antimicrobial peptides alpha and beta defensins, as well as the promotion of epithelial cell survival and proliferation (75, 76). ILC3s and ILC3-derived IL-22 are critical in containing dissemination of commensal bacteria in immune-deficient animals. In the absence of ILC3s, host-derived bacteria of the *Alcaligenes* species disseminate to peripheral organs and induce systemic inflammation (77). IL-22 also acts on intestinal epithelial stem cells, and radio-resistant IL-22-producing ILC3s from the recipient are key to limit the severity of intestinal damage during graft versus host disease (GVHD) (78).

While in the short term IL-22-mediated survival and proliferation of epithelial cells may favor tissue healing and repair, prolonged IL-22 signaling, and sustained epithelial proliferation may drive tumor formation (79). Accordingly, recent evidence has linked colonic ILC3s to colon cancer in a genetically prone bacterial-driven model of colon cancer (80). ILC3s have also been involved in other human disease, such as Crohn’s disease (81) and psoriasis (82).

NCR^−^ ILC3s have been shown to negatively regulate adaptive CD4 T cell responses to commensals. This process does not depend on IL-17 or IL-22 but requires MHC class II expression on ILC3s and, by mechanisms yet to be completely elucidated, restricts CD4 T cell proliferation to commensal antigens (83).

Interestingly, in human tonsil, ILC3s produce GM-CSF, BAFF, and LIF in addition to IL-22 and express high levels of CD40L and RANKL (44). GM-CSF promotes accumulation of granulocyte-monocyte progenitors (GMPs) and mediates immunopathology in a mouse model of T cell-mediated colitis (84). Surprisingly, in steady state conditions, ILC3-derived GM-CSF is essential to induce development of CD103^+^CD11b^+^ DCs that instruct differentiation of FoxP3^+^ Treg cells in an IL-10, TGF-β, and retinoic acid-dependent fashion. ILC3s produce GM-CSF selectively upon stimulation with IL-1β, which is released by macrophages activated by microbial products (85). Selective loss of GM-CSF in ILC3s results in impaired oral tolerance to dietary antigens.

CD40L expression and BAFF production by ILC3s enhance antibody secretion by marginal zone B (MZB) cells (86). Interestingly, ILC3s activate marginal reticular cells (MRCs) by providing TNF-α and lymphotoxin (LT). They also receive survival signals from MRCs, including IL-7. The interaction between MZB cells and ILC3s also involves expression of DLL1 by ILC3s, a Notch ligand that may activate Notch2 on MZB cells (86). Furthermore, in these settings, GM-CSF produced by ILC3s promotes APRIL secretion by neutrophils, boosting their B helper phenotype and further promoting IgM, IgG, and IgA production by MZB cells. RORγt^+^ LTi-like ILC3s induce T cell-independent IgA production in isolated lymphoid follicles (ILFs). The simultaneous stimulation of stromal cells by LTi-like cells, via LT/LTβR, and by bacteria, via TLRs, induces recruitment of DCs and B cells promoting formation of ILFs and boosting T cell-independent IgA production in ILFs (87). Type 3 ILCs have also been shown to control T cell-dependent IgA production through release of soluble LTα3, which induces T cell homing to the gut (88). Ultimately, reduction in IgA levels due to absence or dysfunction of ILC3s in small intestine will induce changes in the microbial communities that may cause immunopathology. Collectively, these studies indicate that ILC3s may provide help to B cell responses at mucosal barriers by multiple mechanisms that may cooperate in providing optimal protection from environmental insults.

The relevance of LIF production and RANKL expression by ILC3s has yet to be understood. However, RANKL has been shown to be essential for the differentiation of the specialized microfold cells (M cells) that overlay the dome region of PP (89). Therefore, RANKL expression by ILC3s could be relevant in the context of PP function and biology and in the transport of particulate antigen from the lumen to APCs located in the sub-epithelial dome area of PP.

Although a transient burst of IL-22 from ILC3s seems to play protective effects in many scenarios and in particular during bacterial infections, a recent study has defied this view. IL-22 induces robust expression of antimicrobial proteins, such as lipocalin and calprotectin. These proteins are known to sequester metal ions including iron, zinc, and manganese. By subtracting these ions to commensal bacteria, IL-22 favors expansion of pathogenic bacteria, such as *Salmonella typhimurium* that are resistant to ion starvation (90). Therefore, in some circumstances, ILC3s may tip the balance in favor of pathogens, rather than protect from their attack.

In addition to sensing cytokines released in the surrounding microenvironment, ILC3s are also sensitive to nutrients. Recent work has shown that vitamin A deficiency results in decreased numbers of ILC3s in the intestine, which increases susceptibility to bacterial infections. However, the same deficiency induces expansion of ILC2s, which protect from nematode infections (91). Moreover, vitamin A intake by pregnant mothers controls the pool of LTi-like CD4^+^ ILC3s in embryos, the size of lymph nodes and PP, and the efficacy of immune responses to viral infections (92). These findings again emphasize the idea that ILCs are exquisitely sensitive to environmental cues and continuously adapt to rapidly changing settings, such as the ones present at mucosal surfaces.

## The Network of Master Regulators of ILC Development

Until recently, the general consensus was that the very same transcription factors that drive Th1, Th2, and Th17 commitment and differentiation also regulate ILC1, ILC2, and ILC3 development, respectively. Moreover, it seemed that cNKs and the other ILC groups significantly differed in their developmental requirement for transcription factors.

**Figure 1:**
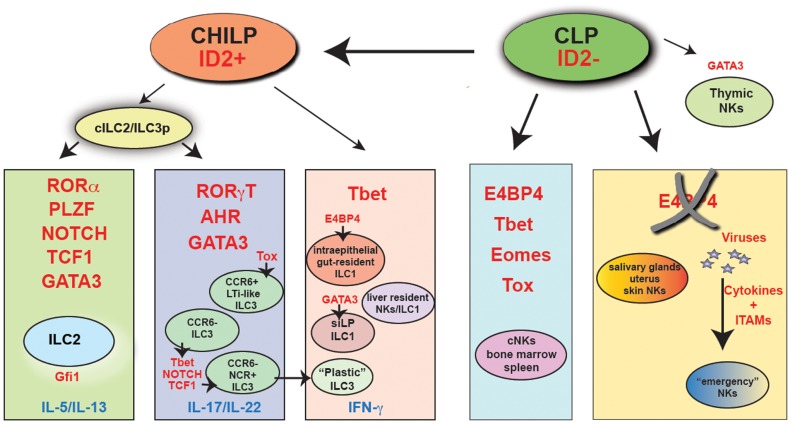
**The major transcriptional pathways that control ILC and NK cell development**. Indicated in red are the key transcription factors that have been shown being absolutely required *in vivo* for the development of each cell subset. “Emergency” NK cells are NK cells that develop in the absence of E4BP4, following viral infection. It is presently unclear whether these cells are Tbet-dependent. CHILP, common helper innate lymphoid precursor; CLP, common lymphoid progenitor; cILC2/ILC3p, common ILC2/ILC3 progenitor.

This concept has been recently challenged by a number of studies suggesting that developmental requirements for NK cell subsets and ILCs are more complex than originally anticipated (Figure 1). Here, we will highlight the most recent progresses in the field.

All ILC groups, including cNKs, depend on Id2 (47, 93). Id2 is a transcriptional repressor that, by blocking members of the E2 family of transcription factors, suppresses the B cell fate potential in a progenitor cells that develop downstream of the common lymphoid precursor (CLP). A recent study that took advantage of a reporter mouse expressing GFP under the control of the Id2 promoter to track Id2-expressing cells has shown that Id2 is not expressed in CLPs. Id2 is, however, expressed at very high level in all ILCs, including cNKs (94). In NK cells, Id2 is not required for the NK cell lineage specification, since development of CD122^+^NK1.1^−^ NK cell progenitors is not impaired in Id2-deficient mice. Nevertheless, Id2 expression in NK cells is required for acquisition of a fully mature phenotype. The few NK cells that develop in the absence of Id2 have a phenotype that closely resemble thymic NK cells, express high levels of CD127 and produce large amounts of IFN-γ (95). In committed B cells, the transcription factor EBF1 mediates Id2 suppression (96). According to the idea that Id2 is globally required for development of all ILCs, EBF1-deficient pro-B cells acquire the capacity to differentiate into T cells and into functionally competent ILC2s and ILC3s (97). Importantly, the generation of an Id2-reporter mouse has allowed the identification of a common ILC progenitor, or CHILP (common “helper-like” ILC lineages progenitor) (10) that can generate CD127^+^NKp46^+^ siLP ILC1, ILC2, and ILC3 *in vivo* and *in vitro*. This Lin^−^Id2^+^ progenitor also expresses CD127 and the integrin α4β7 but does not express CD25 (which is present on differentiated ILC2) or CD122, a marker of NK cell precursors. Intriguingly, this precursor is only slightly affected by IL-7 deficiency and can generate liver-resident CD49a^+^Eomes^−^ NK cells, but not cNKs (10). Further confirming the existence of a common ILC progenitor, an other recent study performing fate-mapping experiments shows that ILCs, including intraepithelial ILC1, ILC2, and NCR^+^ ILC3, derive from a common committed precursor present in fetal liver and adult bone marrow that transiently expresses the transcription factor PLZF (encoded by *Zbtb16*) (98). This transcription factor was known to be required for NKT development, but it is dispensable for cNK cells and CD4^+^ LTi-like cells (99). Reconstitution experiments with *Zbtb16*-deficient or -sufficient bone marrow cells in lethally irradiated mice indicated that *Zbtb16* is absolutely required for ILC2s development only. On the contrary, it seems to be dispensable for NCR^+^ ILC3s reconstitution, despite its high expression in this subset. As *Zbtb16* is controlled by Id2 (100), the PLZF-expressing common ILC precursor may be downstream of the Id2^+^CD127^+^CD25^−^α4β7^+^ CHILP.

Most likely downstream of the CHILP, all ILC3 subsets require the Th17 master regulator RORγt (101, 102). In the absence of RORγt, Lti, and Lti-like cells fail to develop and RORγt-deficient mice do not possess lymph nodes, PP, cryptopaches (CP) nor ILFs (103, 104), which derive from CP under signals coming from the microbiota through the nod-like receptor (NLR) NOD1 (105). ILC3s also require aryl hydrocarbon receptor (AHR) (106–108), which drives IL-22 secretion in Th17 cells (109, 110). In the absence of AHR, both Lti-like ILC3s and NCR^+^ ILC3s are severely diminished. In addition, small intestine CP and ILFs are absent, while colonic patches develop normally (111). Some studies have suggested that exogenously provided AHR agonists, such as tryptophan derivatives added to the diet, play a role in expanding and/or maintaining ILC3s (106, 112). Recent work has also shown that AHR agonists derived from commensal bacteria, such as lactobacilli, increase IL-22 production by ILC3s in the context of a tryptophan rich diet and this, in turn, confers resistance to infection by the opportunistic fungus *Candida albicans* (113). It is also possible that endogenous AHR ligands, such as kynurenine, produced by the enzyme tryptophan dioxygenase (TDO), may play a critical role in ILC3 biology (114).

Unexpectedly, AHR, in ILC3 cells, restrains adaptive Th17 responses. In AHR-deficient mice that lack ILC3s, Th17 cells in siLP undergo an uncontrolled expansion that leads to tissue damage. This Th17 proliferation is due to a dysregulation in the intestinal microbial niche that allows excessive growth of *segmented filamentous bacteria* (SFB), commensals known to drive Th17 differentiation (115).

In addition to RORγt and AHR, ILC3s also depend on Notch signaling for their development (111, 116). In RBP-J_k_^−/−^ mice that lack all Notch signaling, NCR^+^ ILC3s are selectively reduced. Notch also promotes RORγt^+^ ILC differentiation from adult bone marrow precursors (117). Recent studies have also shown that NCR^+^ ILC3s, which develop post-natally and reach maximal expansion 4–6 weeks after birth in mouse small intestine LP (118), express Tbet (encoded by *Tbx21*) and require Tbet for development (46, 116, 119, 120). Induced by IL-23 and cues from the microbiota, Tbet is essential to instruct IFN-γ production and protection from pathogens such as *S. typhimurium* (46). Tbet expression seems to induce Notch, as Tbet haplo-insufficiency results in reduced Notch expression (116). However, it is conceivable that AHR and Tbet may cooperate in inducing Notch and final maturation of NCR^+^ ILC3s.

The requirement for Tbet in NCR^+^ ILC3s is somewhat surprising since human tonsil ILC3s do not express Tbet *ex vivo* (44). In fact, in steady state conditions, Tbet expression is limited to ILC1s and cNKs, while RORγt is selectively expressed by ILC3 (9).

Human tonsil ILC3s also express high levels of CCR6 and can easily be sorted at high purity based on NKp44 and CCR6 expression (44, 121), while mouse NCR^+^ ILC3s are CCR6 negative (46). Whether the discrepancies between the mouse and human system are linked to tissue specific differences (intestine versus tonsil) still remains to be determined. It is conceivable that Tbet may be needed at later stages of ILC3s differentiation, which may be poorly represented in the tonsil. In agreement with this hypothesis, tonsil ILC3s are characterized by an intrinsic plasticity and, when stimulated with cytokines such as IL-2 and IL-15 *in vitro*, can generate IFN-γ producing pro-inflammatory cells that acquire some features of cNK (8, 44). Specific engagement of cell surface receptors expressed by ILC3s by cognate ligands present in some tissues but not in others may also be an issue. Indeed, a recent study has suggested that stimulation of ILC3s via the DAP12-associated activating receptor NKp44 induces production of TNF-α and convert them to a more inflammatory-prone cell type (122). Experiments in mice have suggested that ILC3 conversion to an IFN-γ/TNF-α secreting pro-inflammatory cell type can occur *in vivo* (45). Moreover, recent fate-mapping experiments, aimed to track cells that expressed RORγt at any stage of their life cycle, show that “plastic” or “converted” ILC3s are present within the RORγt^−^NKp46^+^NK1.1^+^ population in siLP and cluster with siLP ILC1s and cNK (10).

Finally, STAT3 expression in ILC3s is required for robust IL-22 production and protection by intestinal infection with *C. rodentium* (123).

Group 2 ILCs necessitate RORα (124), Gfi1 (125), and Gata-3 for their development (94, 125–127). Gfi1 specifically controls the response to IL-33 and TSLP and the production of IL-5, but not IL-13, in ILC2s (125). The requirement for Gata-3 by ILC2s closely resembles the need for Gata-3 in Th2 differentiation. However, Gata-3 is also necessary for thymic NK cell development (26). Moreover, a recent study has indicated that Gata-3 is required for the generation of ILC3s (128), before the identification of the CHILP (10). Transfer of Gata-3-deficient fetal liver precursor cells in mice lacking ILCs prevents reconstitution of all RORγt^+^ ILC3s. This finding has led to the hypothesis that a common precursor might exist between ILC2s and ILC3s (128). In agreement with this view, another transcription factor TCF-1 (encoded by Tcf7) is required for ILC2s and NCR^+^ ILC3s (129, 130). TCF-1 in ILC2s acts downstream of Notch and forced expression of TCF-1 can bypass Notch requirement for ILC2 generation. TCF-1 also induces Gata-3, which is responsible for upregulation of *Il-17rb* (the receptor for IL-25) and *Il2ra* (CD25) in developing ILC2s. However, in the absence of Gata-3, TCF-1 directly controls the expression of *Il7r* (CD127) (130). In NCR^+^ ILC3s, TCF-1 acts downstream of Tbet and Notch to promote ILC3 development (129).

Gata-3 is also required to induce differentiation and/or maintenance of siLP ILC1s (10), as deletion of Gata-3 in NKp46^+^ cells results in a dramatic reduction of this ILC subset, while does not affect ILC3s or cNKs. In addition, early deletion of Gata-3 in hematopoietic cells abolishes the development of all CD127^+^ ILCs (131).

While it is not clear whether TCF-1 is also required for siLP ILC1s, the data available suggest that some transcription factors may be required at multiple stages of ILC differentiation and may be turned on and off at different transitional steps with a hierarchy that is still poorly defined.

Intraepithelial ILC1s and cNKs cells share some requirements in transcription factors for their differentiation. To begin, both cell populations require Tbet for development (9, 132). In addition, Tbet is necessary for the development of liver-resident NK cells that express Trail, CD49a, lack DX5, and Eomes (133) and have features of tissue-resident memory cells based on parabiosis experiments (30, 134). Although these cells were originally thought to represent an NK immature cell subset (135), more recent data suggest that they represent a distinct lineage of NK cells that share part of their transcriptional program with NKT cells (133). While it is not clear whether intraepithelial ILC1s derive from the CHILP, liver-resident NK cells could represent a true subset of ILC1s related to the siLP ILC1s, since they appear to differentiate from the CHILP (10).

Intraepithelial ILC1s and cNK cells also require E4BP4 (encoded by *Nfil3*) to develop (9, 136, 137). E4BP4 in NK cell development was previously thought to act directly downstream of IL-15R, which is required for NK cell maintenance (138, 139). However, more recent data suggest that viral infections can rescue NK cell development independently of E4BP4 in a process that requires inflammatory cytokines such as type 1 interferon and IL-12 along with ITAM signaling (140). E4BP4-independent NK cells are functional and persist in the periphery in an IL-15 dependent fashion. Ablation of E4BP4 after the earlier steps of NK cell commitment completely bypasses the requirement for E4BP4 in NK cell development (140). In addition, E4BP4 is less stringently required for NK cell development outside of the bone marrow. Liver-resident Trail^+^, DX5^−^ NK cells develop in the absence of E4BP4, especially when T cell competition is eliminated (30, 141, 142). Salivary gland NKs also do not require E4BP4 (29). The role for E4BP4 in thymic NK cell development is still controversial. Thymic NK cells seem to develop normally *in vitro* from E4BP4-deficient double negative (DN)1 and DN2 thymocytes (141). However, a second study indicates that thymic NKs *in vivo* develop along an E4BP4-dependent pathway (142). According to this report, E4BP4 is necessary to enforce Eomes expression in cNKs as well as in thymic NKs. Further confirming that E4BP4 may be necessary to drive Eomes expression, another report demonstrates that E4BP4 is strictly required for the transition from CLP to NK cell progenitor and regulates Eomes and Id2 expression in NK cells (143). Challenging the previous concept that E4BP4 acts downstream of the IL-15R (136), this study shows that E4BP4 operates upstream of IL-15 signaling and in the absence of E4BP4 CD122^+^ NK precursors fail to develop. E4BP4 expression is detected earlier that Tbet, Eomes, or Id2 expression and chromatin immunoprecipitation experiments demonstrate that E4BP4 promotes transcription of Eomes and Id2 by binding directly to the regulatory regions of their genes (143). These data are, however, in conflict with the study by Seillet et al., which shows that Id2 expression is normal in the absence of E4BP4 (142). Further studies will be necessary to elucidate the exact hierarchy in which transcription factors need to be expressed in order to grant successful development and maturation of NK cells and ILCs.

Finally, cNK cells share with CD4^+^ LTi-like cells the requirement for the HGM-box transcription factor superfamily member TOX. TOX-deficient animals lack NK cells and do not develop lymph nodes nor PP (144). Although it has been hypothesized that TOX controls induction or maintenance of Id2 in precursor cells, the exact mechanisms by which TOX regulates development of cNKs and LTi-like cells and whether it is necessary for other ILC subsets is unclear.

## Concluding Remarks

Studies over the past few years have indicated that adaptive immunity has adopted modules already established in the innate branch of the immune system to counteract pathogens and to maintain homeostasis at mucosal surfaces, which are constantly exposed to environmental insults and non-self-microbial communities. The identification of innate subsets that mimic Th1, Th2, and Th17/Th22 Thelper cells has greatly advanced our understanding of how immune responses are orchestrated and shaped. Future challenges in the field will be to understand whether additional subsets of ILCs are present that prevent or control autoimmune processes, i.e., whether an innate functional equivalent of regulatory T cells is in place. In addition, it will be important to delineate whether dedicated subsets of APCs or stromal cells are located in mucosal tissues that can preferentially activate a particular ILC group, or whether this activation process depends on specific signals provided by the intruder. Elucidating whether diverse inflammatory milieus can elicit different responses and immunological outcomes from the same ILC group will be also important. In the long term, a better understanding of the very first events that take place early on during the initiation of an immune response may be key in designing better strategies for vaccine development and therapeutic intervention.

## Conflict of Interest Statement

The authors declare that the research was conducted in the absence of any commercial or financial relationships that could be construed as a potential conflict of interest.
